# Patience

**Published:** 1890-05-03

**Authors:** 


### Words of Consolation.

### PATIENCE.

We have seen that pain and suffering, though not always a punishment for sin, are nevertheless necessary for our perfection in the sight of God. Let us consider, then, in what way we should receive the crosses in our lot, and what frame of mind we ought to cultivate, in order to gain the full benefit of our correction.

We must first realize that our Blessed Lord, whose life we should copy, came down from Heaven and took upon Him our miseries, not because He was obliged to do so, but out of pure love to men. From the hour of His birth until His death, He endured with sweetness, disgrace, revilings, and ingratitude from those whom He was loading with benefits. If He, then, bore unmerited suffering with patience, ought not we for the welfare of our souls to bear our crosses cheerfully? Life may seem tedious by reason of our labour and sorrow, yet Christ's gracious example and the footsteps of the saints have made the way bright and clear and endurable even to the weak. Christ will show us the right path to His eternal kingdom if we ask Him in faith. If He had not gone before and taught us, how few would have cared to tread the narrow way, for, notwithstanding His noble example, we are, alas! careless and lukewarm.

To gain a calm peace of mind, we should not dwell on our own sufferings, but compare them with those of others, who have been more strongly tempted, or grievously afflicted than ourselves, and have been tried and exercised in many ways. By this means our own trials may be easier to bear.

If they still seem great and burdensome, then beware lest impatience be the cause, for whether they be great or whether they be small all should be undergone with patience.

That person is not truly patient who is willing to suffer only so much as he thinks good, and from whom he pleases. He will say to himself "I ought not to endure things of this sort," or "why does such a one reproach me with things I never thought of? Some things I might deserve but not that." Oh! thou whom thus thinkest, remember Him who bare the contradiction of sinners without murmuring, and take indifferently from every creature, whatever befals thee, with thankfulness as if from the hand of God. The smallest thing, if suffered for God's sake, will not lose its reward.

If thou desirest victory be always prepared for the fight, for without a combat thou canst not attain unto the crown of patience. If thou art unwilling to suffer, thou refusest to be crowned. But if thou desirest to be crowned, fight manfully, endure patiently; without labour there is no arriving at rest, nor without fighting can the victory be reached. "The trying of your faith worketh patience," says S. James, and adds "Let patience have her perfect work, that ye may be perfect and entire wanting nothing." It was those who had kept the word of patience in the Church of Philadelphia whom our Lord promised to keep from the hour of temptation.

